# Supplementary material for: The barley mutant multiflorus2.b reveals quantitative genetic variation for new spikelet architecture
Source: Theor Appl Genet. 2021 Nov 13;135(2):571–90. doi: 10.1007/s00122-021-03986-w (PMC8866347; doi:10.1007/s00122-021-03986-w)
Supplement: Supplementary file 1 — Supplementary file1 (PPTX 63172 KB) [file 122_2021_3986_MOESM1_ESM.pptx]

## Slide 1
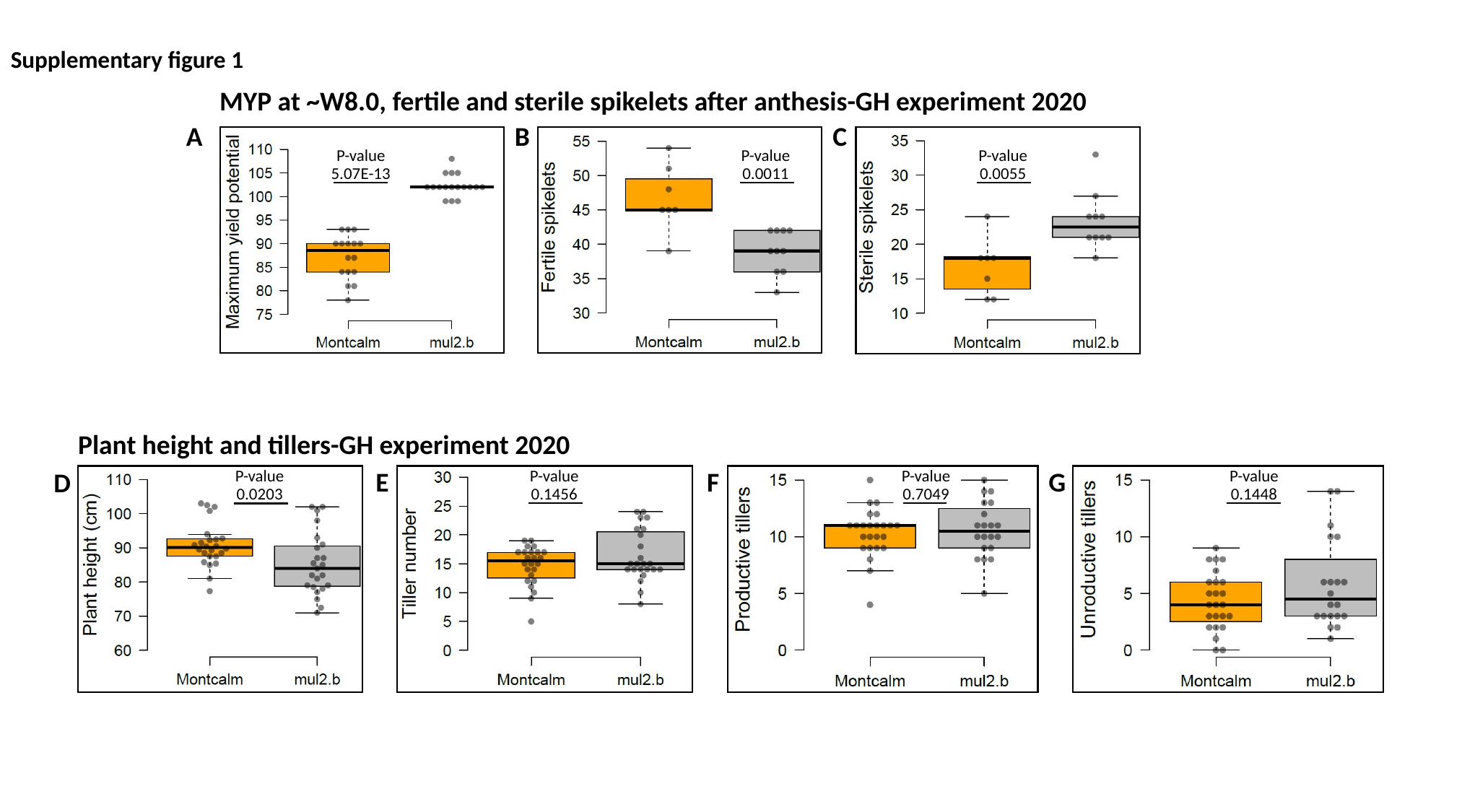

Supplementary figure 1
MYP at ~W8.0, fertile and sterile spikelets after anthesis-GH experiment 2020
A
B
C
P-value
5.07E-13
P-value
0.0011
P-value
0.0055
Plant height and tillers-GH experiment 2020
E
F
G
D
P-value
0.1456
P-value
0.7049
P-value
0.1448
P-value
0.0203

## Slide 2
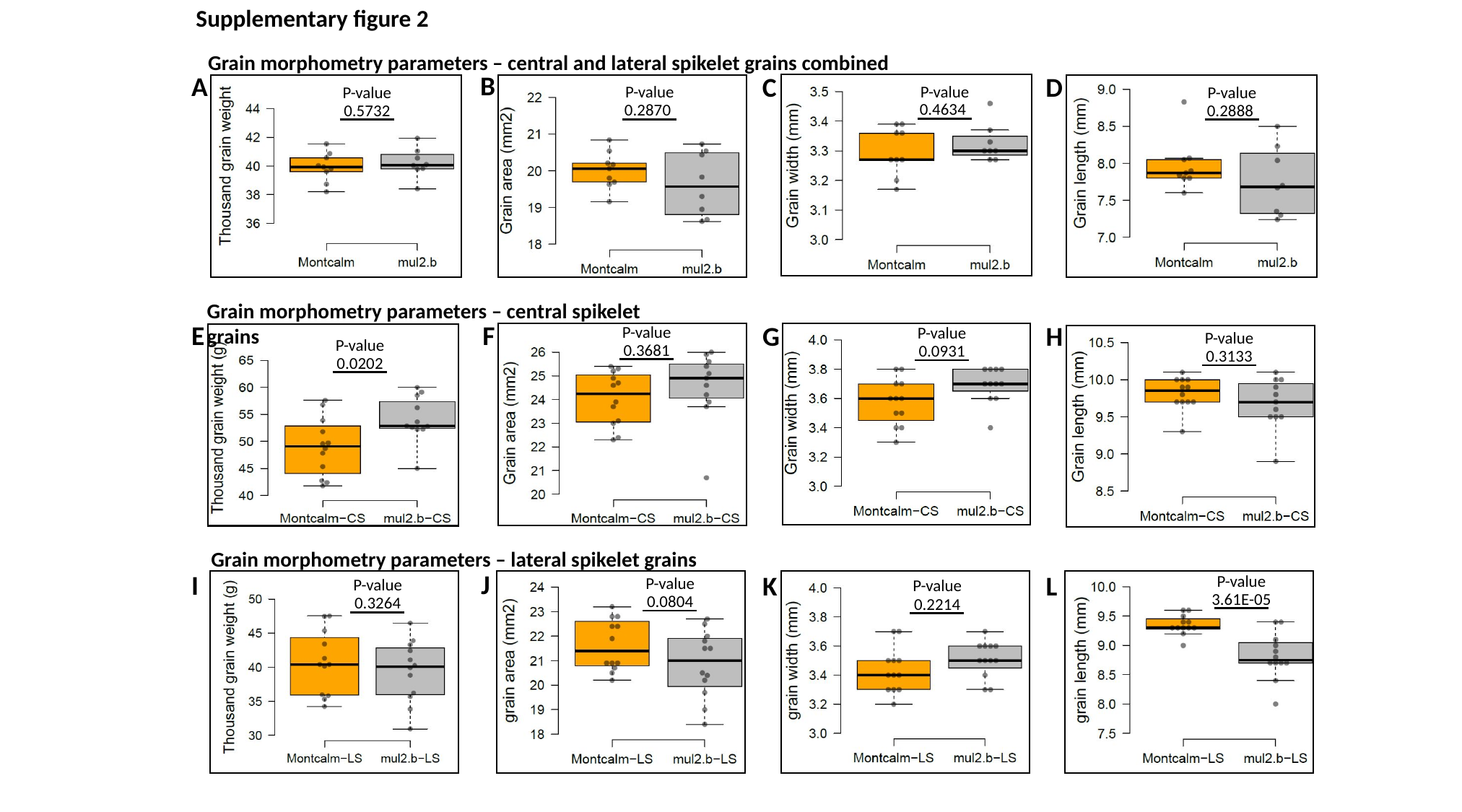

Supplementary figure 2
Grain morphometry parameters – central and lateral spikelet grains combined
B
A
C
D
P-value
0.4634
P-value
0.2870
P-value
0.5732
P-value
0.2888
Grain morphometry parameters – central spikelet grains
F
E
G
H
P-value
0.3681
P-value
0.0931
P-value
0.3133
P-value
0.0202
Grain morphometry parameters – lateral spikelet grains
J
I
K
L
P-value
3.61E-05
P-value
0.0804
P-value
0.3264
P-value
0.2214

## Slide 3
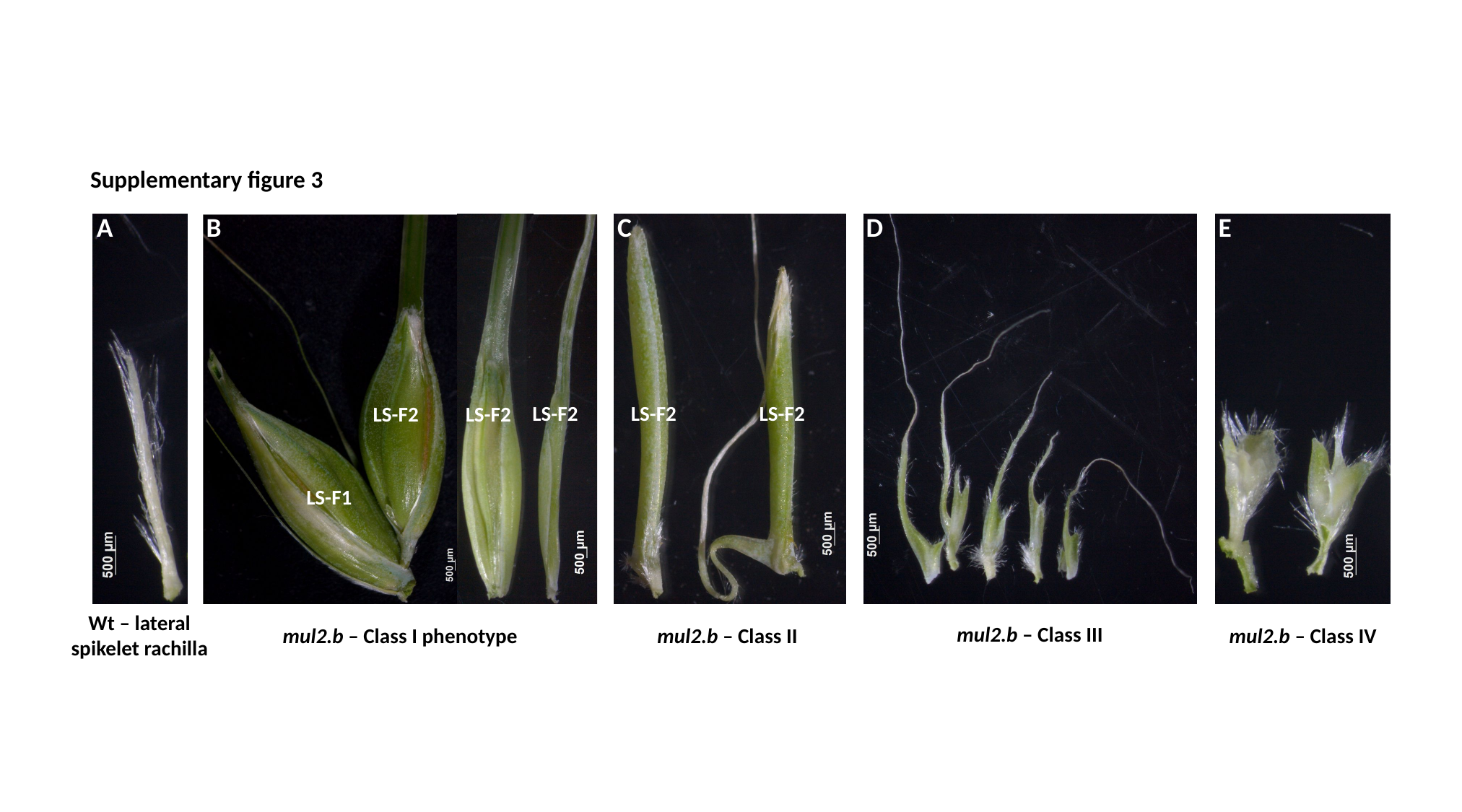

Supplementary figure 3
A
B
C
D
E
LS-F1
Wt – lateral spikelet rachilla
mul2.b – Class III
mul2.b – Class I phenotype
mul2.b – Class II
mul2.b – Class IV
LS-F2
LS-F2
LS-F2
LS-F2
LS-F2

## Slide 4
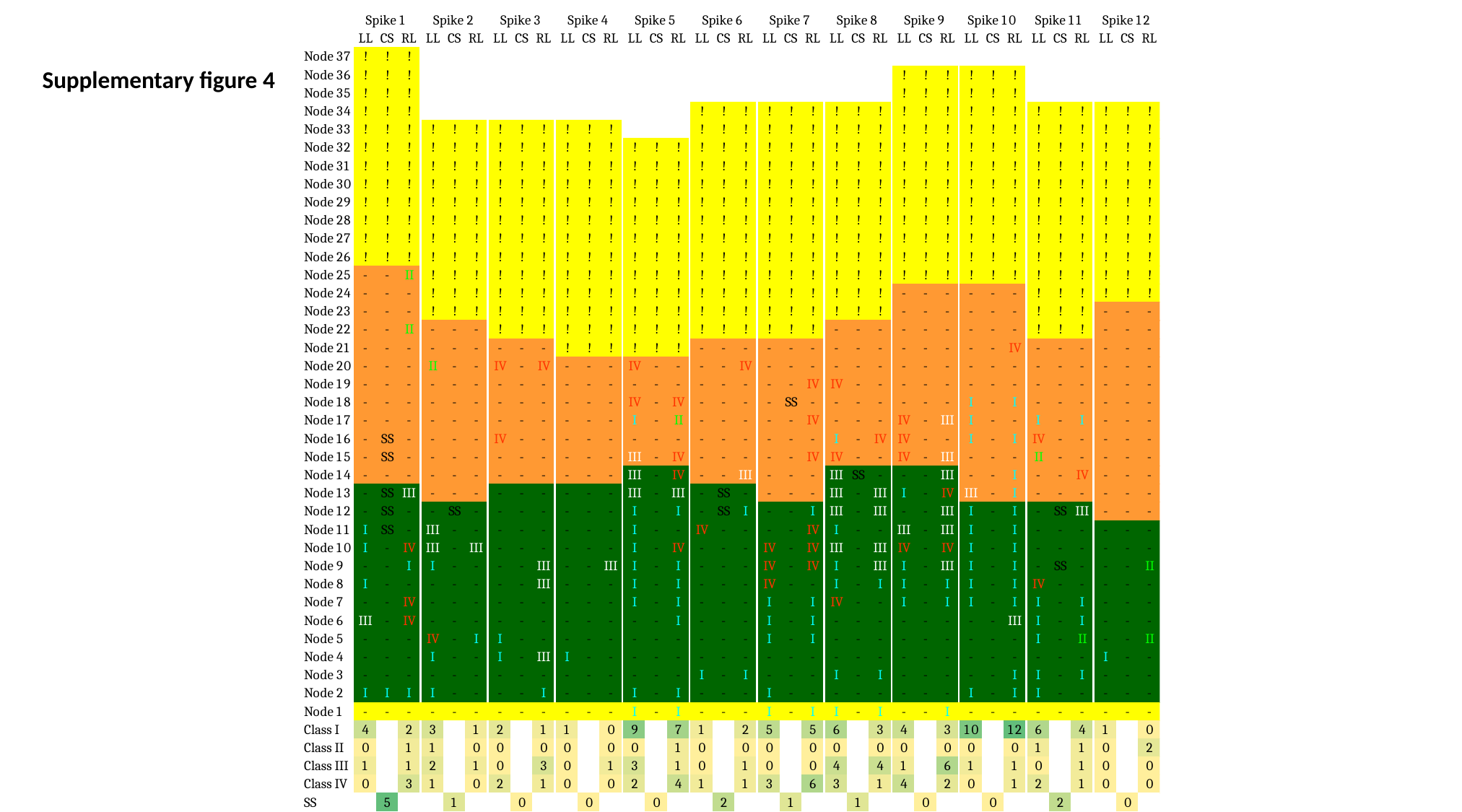

Supplementary figure 4

## Slide 5
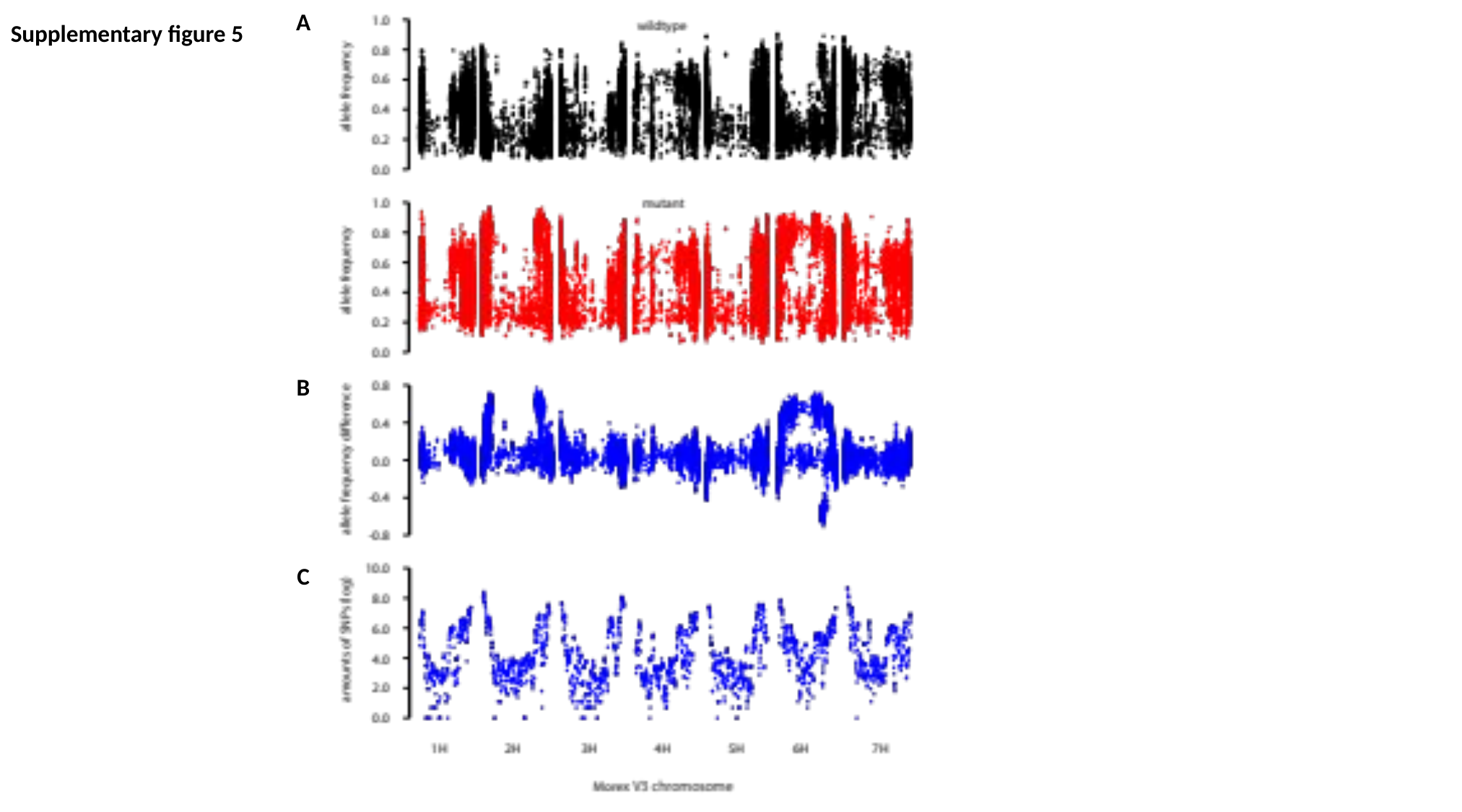

A
B
C
Supplementary figure 5

## Slide 6
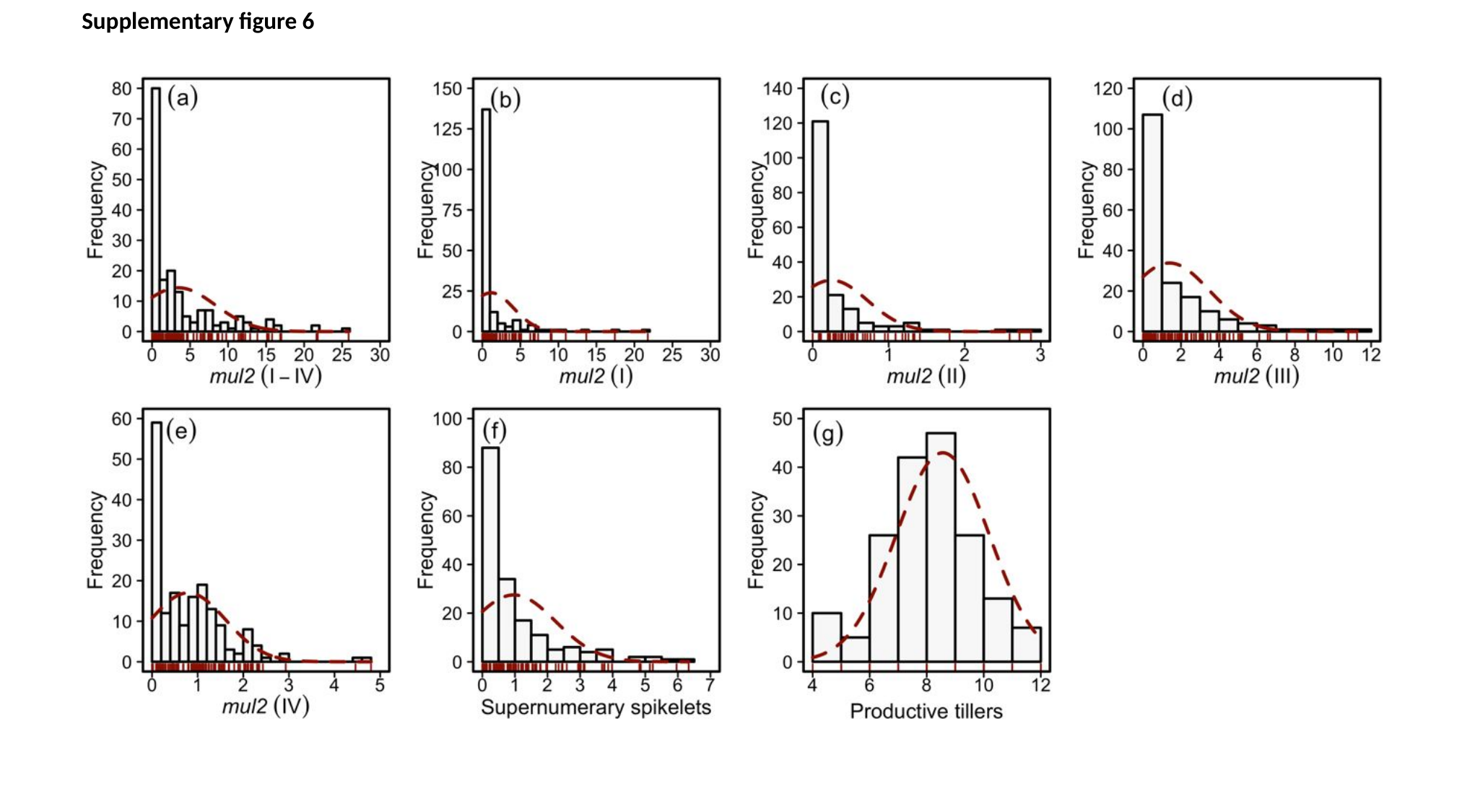

Supplementary figure 6

## Slide 7
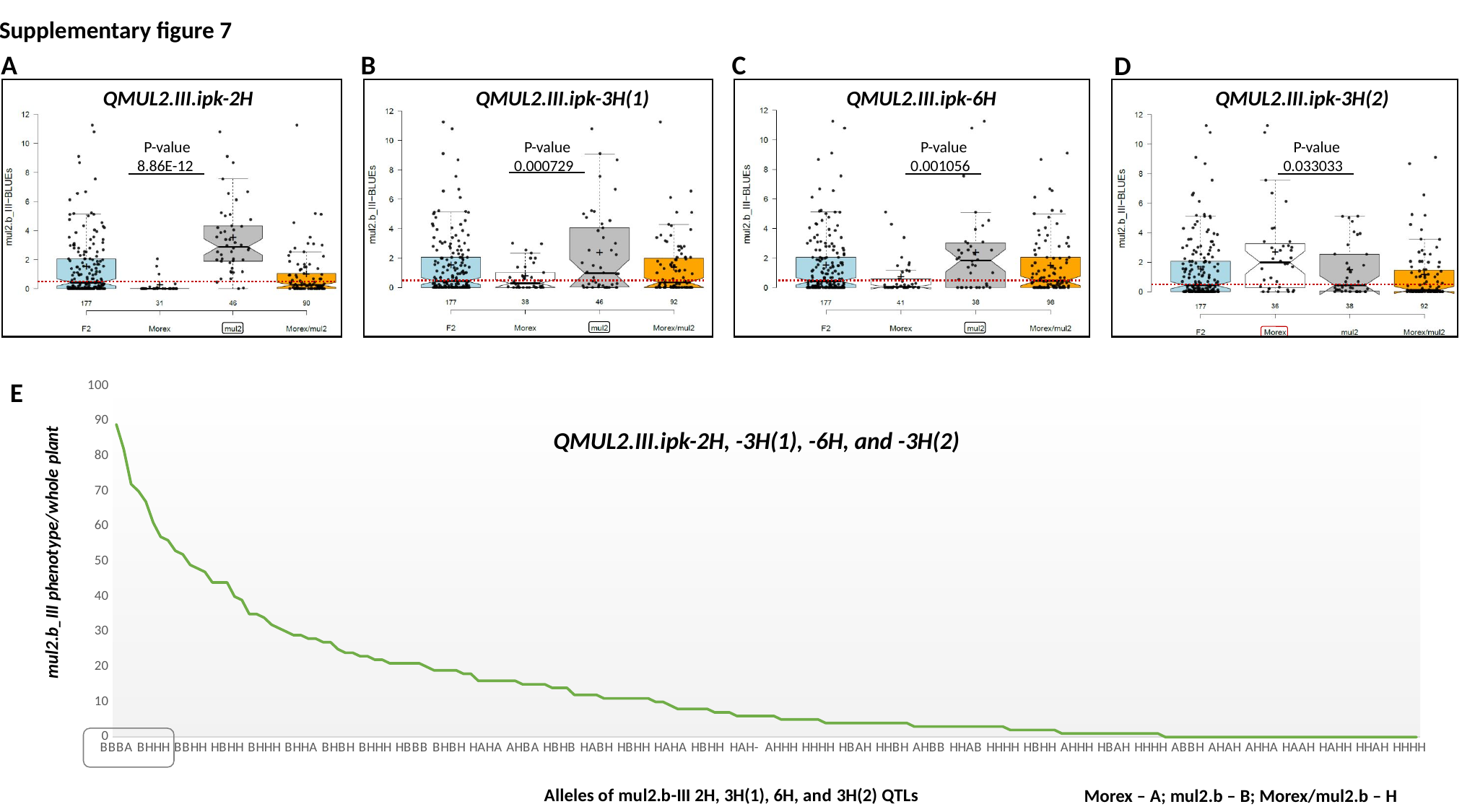

Supplementary figure 7
A
B
C
D
QMUL2.III.ipk-2H
QMUL2.III.ipk-3H(1)
QMUL2.III.ipk-6H
QMUL2.III.ipk-3H(2)
P-value
8.86E-12
P-value
0.000729
P-value
0.001056
P-value
0.033033
E
### Chart
| Category | mul2_II |
|---|---|
| BBBA | 89.0 |
| HHBA | 82.0 |
| BBHH | 72.0 |
| BBBA | 70.0 |
| BBHH | 67.0 |
| BHHH | 61.0 |
| BHHA | 57.0 |
| BBHA | 56.0 |
| BBHB | 53.0 |
| BBHA | 52.0 |
| BBHH | 49.0 |
| HHAB | 48.0 |
| BBHB | 47.0 |
| BHAA | 44.0 |
| BHBB | 44.0 |
| HBHH | 44.0 |
| BBHB | 40.0 |
| BHBB | 39.0 |
| BBHA | 35.0 |
| BHAA | 35.0 |
| BHHH | 34.0 |
| H-BA | 32.0 |
| HBBH | 31.0 |
| HHBA | 30.0 |
| BABH | 29.0 |
| BHHA | 29.0 |
| BBHB | 28.0 |
| BHHB | 28.0 |
| BHHA | 27.0 |
| HHBH | 27.0 |
| BHBH | 25.0 |
| BAHA | 24.0 |
| HHBA | 24.0 |
| BAB- | 23.0 |
| BABB | 23.0 |
| BHHH | 22.0 |
| HBHA | 22.0 |
| BAHH | 21.0 |
| BAHH | 21.0 |
| BBHH | 21.0 |
| HBBB | 21.0 |
| HHBH | 21.0 |
| BHHA | 20.0 |
| AHHA | 19.0 |
| BHAH | 19.0 |
| BHBH | 19.0 |
| HBHA | 19.0 |
| BHBB | 18.0 |
| HHHH | 18.0 |
| BHHH | 16.0 |
| HAHA | 16.0 |
| HBHH | 16.0 |
| HBHH | 16.0 |
| HHAH | 16.0 |
| HHB- | 16.0 |
| AHBA | 15.0 |
| BHAB | 15.0 |
| HHAH | 15.0 |
| HHBA | 15.0 |
| BHAH | 14.0 |
| HBHB | 14.0 |
| HHHH | 14.0 |
| BHAH | 12.0 |
| BHH- | 12.0 |
| BHHH | 12.0 |
| HABH | 12.0 |
| AABH | 11.0 |
| BAHH | 11.0 |
| BHBB | 11.0 |
| HBAH | 11.0 |
| HBHH | 11.0 |
| HBHH | 11.0 |
| HHBH | 11.0 |
| HBHB | 10.0 |
| HHHA | 10.0 |
| HAHA | 9.0 |
| BAHB | 8.0 |
| BHHB | 8.0 |
| HAHA | 8.0 |
| HAHH | 8.0 |
| HBHH | 8.0 |
| HAAH | 7.0 |
| -HH- | 7.0 |
| HHBH | 7.0 |
| BAHB | 6.0 |
| HAH- | 6.0 |
| HAHH | 6.0 |
| HBAB | 6.0 |
| HHH- | 6.0 |
| HHHB | 6.0 |
| AHHH | 5.0 |
| HAH- | 5.0 |
| HBBB | 5.0 |
| HHHA | 5.0 |
| HHHB | 5.0 |
| HHHH | 5.0 |
| AHHH | 4.0 |
| HAAH | 4.0 |
| HAHH | 4.0 |
| HBAB | 4.0 |
| HBAH | 4.0 |
| HBBB | 4.0 |
| HBHH | 4.0 |
| HHA- | 4.0 |
| HHAB | 4.0 |
| HHBH | 4.0 |
| HHHA | 4.0 |
| HHHB | 4.0 |
| ABAH | 3.0 |
| ABHA | 3.0 |
| AHBB | 3.0 |
| BAHB | 3.0 |
| HAHA | 3.0 |
| HBAA | 3.0 |
| HBH- | 3.0 |
| HHAB | 3.0 |
| HHAH | 3.0 |
| HHBB | 3.0 |
| HHHB | 3.0 |
| HHHH | 3.0 |
| HHHH | 3.0 |
| AHHB | 2.0 |
| AHHH | 2.0 |
| BAAH | 2.0 |
| HAHH | 2.0 |
| HBHH | 2.0 |
| HHAH | 2.0 |
| HHBB | 2.0 |
| AHAA | 1.0 |
| AHHA | 1.0 |
| AHHH | 1.0 |
| HAAH | 1.0 |
| HAHA | 1.0 |
| HAHA | 1.0 |
| HAHH | 1.0 |
| HBAH | 1.0 |
| HBHH | 1.0 |
| HBHH | 1.0 |
| HHAB | 1.0 |
| HHBH | 1.0 |
| HHHH | 1.0 |
| HHHH | 1.0 |
| AAAH | 0.0 |
| AAHB | 0.0 |
| ABAH | 0.0 |
| ABBH | 0.0 |
| ABHH | 0.0 |
| ABHH | 0.0 |
| ABHH | 0.0 |
| AHAB | 0.0 |
| AHAH | 0.0 |
| AHAH | 0.0 |
| AHBH | 0.0 |
| AHBH | 0.0 |
| AHH- | 0.0 |
| AHHA | 0.0 |
| AHHB | 0.0 |
| AHHH | 0.0 |
| AHHH | 0.0 |
| HAAH | 0.0 |
| HAAH | 0.0 |
| HAAH | 0.0 |
| HABH | 0.0 |
| HABH | 0.0 |
| -HAH | 0.0 |
| HAHH | 0.0 |
| HAHH | 0.0 |
| HBAH | 0.0 |
| HHA- | 0.0 |
| HHAB | 0.0 |
| HHAH | 0.0 |
| HHAH | 0.0 |
| -HHH | 0.0 |
| -HHH | 0.0 |
| HHHA | 0.0 |
| HHHH | 0.0 |
| HHHH | 0.0 |QMUL2.III.ipk-2H, -3H(1), -6H, and -3H(2)
Morex – A; mul2.b – B; Morex/mul2.b – H

## Slide 8
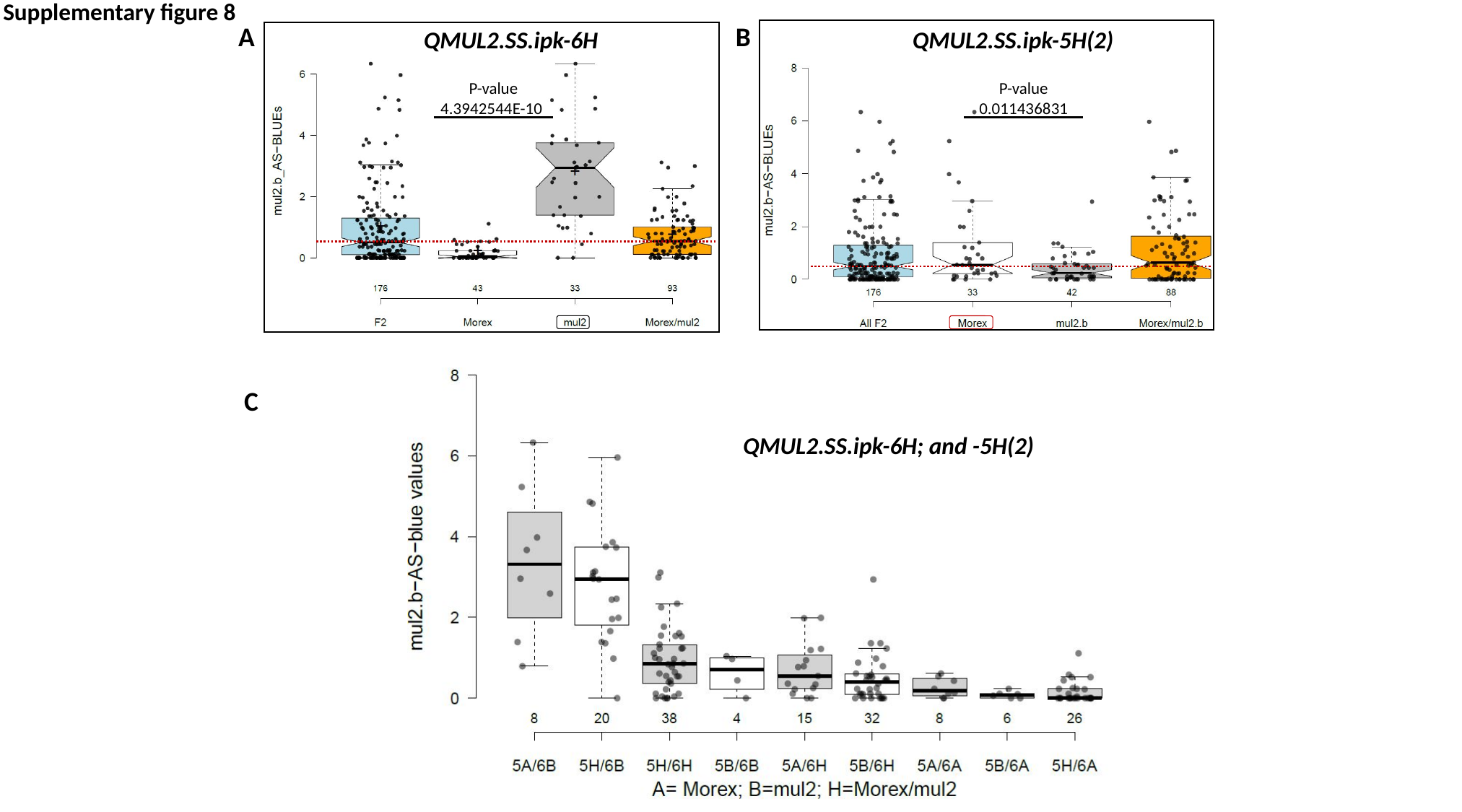

Supplementary figure 8
A
B
QMUL2.SS.ipk-6H
QMUL2.SS.ipk-5H(2)
P-value
4.3942544E-10
P-value
0.011436831
QMUL2.SS.ipk-6H; and -5H(2)
C

## Slide 9
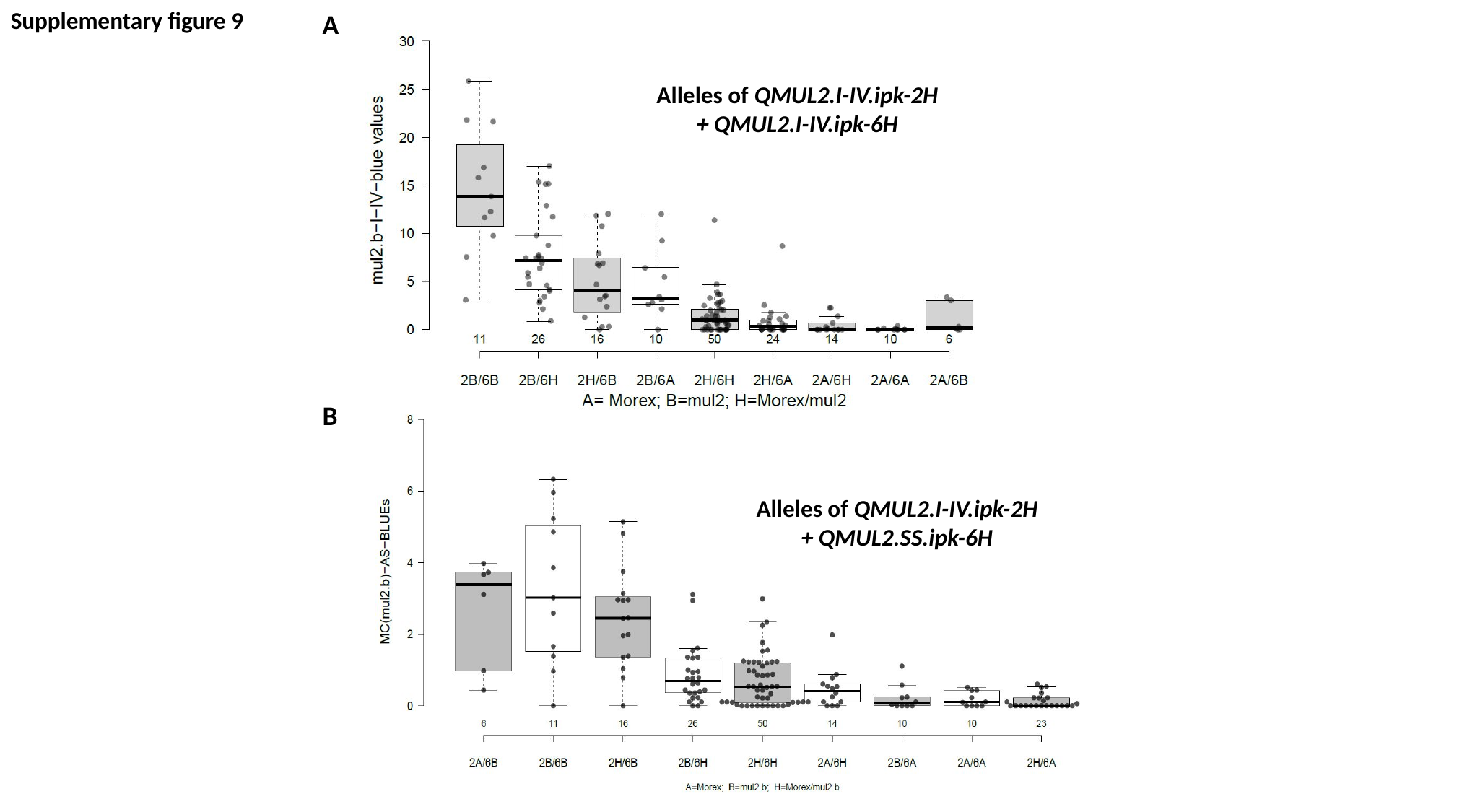

Supplementary figure 9
A
Alleles of QMUL2.I-IV.ipk-2H + QMUL2.I-IV.ipk-6H
B
Alleles of QMUL2.I-IV.ipk-2H + QMUL2.SS.ipk-6H

## Slide 10
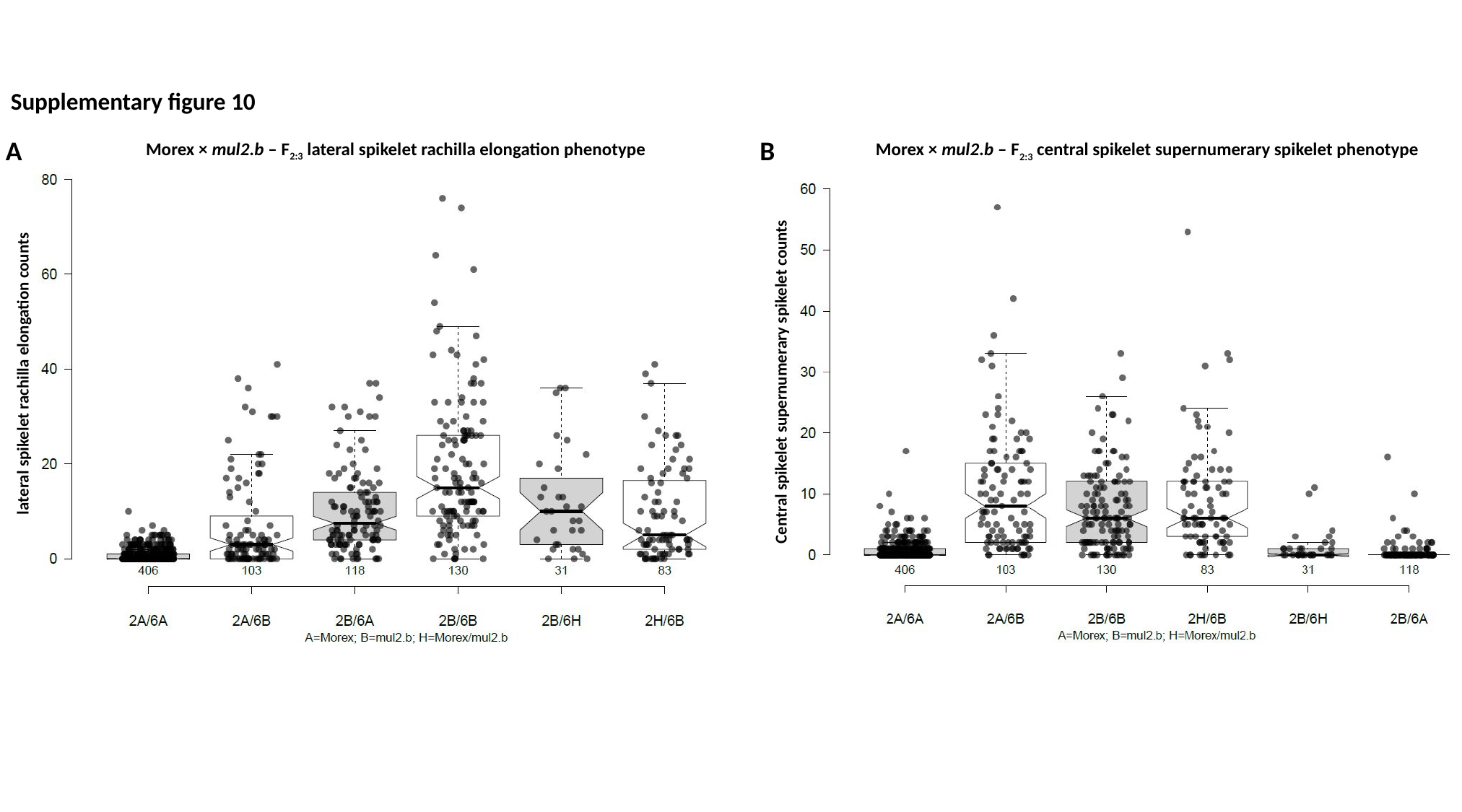

Supplementary figure 10
B
A
Morex × mul2.b – F2:3 lateral spikelet rachilla elongation phenotype
Morex × mul2.b – F2:3 central spikelet supernumerary spikelet phenotype
lateral spikelet rachilla elongation counts
Central spikelet supernumerary spikelet counts

## Slide 11
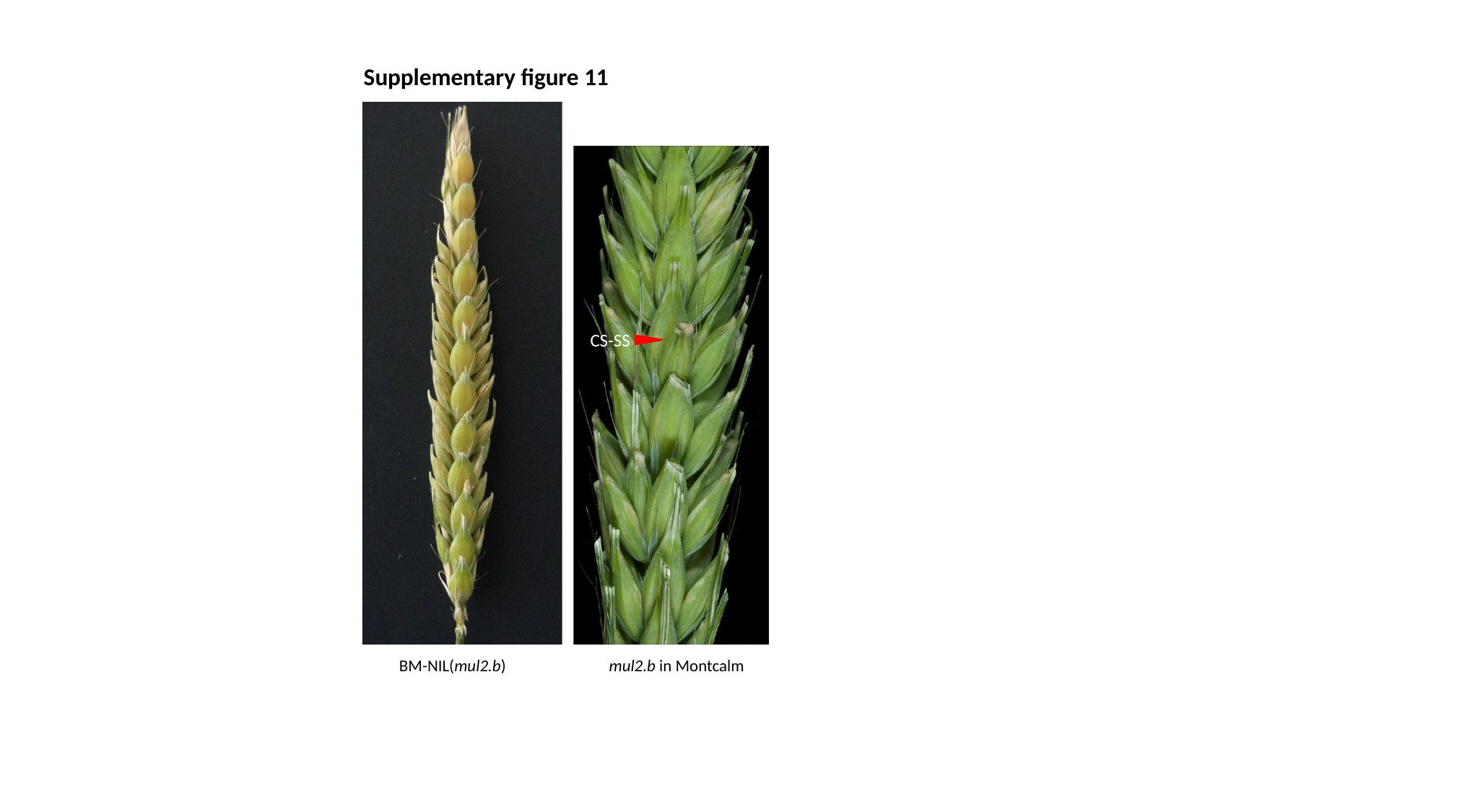

Supplementary figure 11
mul2.b in Montcalm
CS-SS
BM-NIL(mul2.b)
